# Gas exchange mechanisms in preterm infants on HFOV – a computational approach

**DOI:** 10.1038/s41598-018-30830-x

**Published:** 2018-08-29

**Authors:** Christian J. Roth, Kai M. Förster, Anne Hilgendorff, Birgit Ertl-Wagner, Wolfgang A. Wall, Andreas W. Flemmer

**Affiliations:** 1 Institute for Computational Mechanics, Technical University of Munich, 85748 Garching, Germany; 2 Division of Neonatology, Dr. von Hauner Children’s Hospital, Perinatal Center Grosshadern, LMU-Munich, 81337 Munich, Germany; 3 Comprehensive Pneumology Center, Helmholtz Zentrum München, Munich, Germany, Member of the German Lung Research Center (DZL), Munich, Germany; 4 Department of Radiology, LMU-Munich, 81377 Munich, Germany

## Abstract

High-frequency oscillatory ventilation (HFOV) is a commonly used therapy applied to neonates requiring ventilatory support during their first weeks of life. Despite its wide application, the underlying gas exchange mechanisms promoting the success of HVOF in neonatal care are not fully understood until today. In this work, a highly resolved computational lung model, derived from Magnetic Resonance Imaging (MRI) and Infant Lung Function Testing (ILFT), is used to reveal the reason for highly efficient gas exchange during HFOV, in the preterm infant. In total we detected six mechanisms that facilitate gas exchange during HFOV: (i) turbulent vortices in large airways; (ii) asymmetric in- and expiratory flow profiles; (iii) radial mixing in main bronchi; (iv) laminar flow in higher generations of the respiratory tract; (v) pendelluft; (vi) direct ventilation of central alveoli. The illustration of six specific gas transport phenomena during HFOV in preterm infants advances general knowledge on protective ventilation in neonatal care and can support decisions on various modes of ventilatory therapy at high frequencies.

## Introduction

Preterm infants with structurally and functionally immature lungs often require prolonged assisted mechanical ventilation and deserve the most protective methods to reduce the side effects of ventilation therapy. In clinical practice high frequency oscillatory ventilation (HFOV), i.e. a combination of positive end expiratory pressure (PEEP) with superimposed oscillations using very low tidal volumes (*V*_*T*_) at high frequencies (*f*), has been shown to result in superior scores of short-term oxygenation^1^ and long-term lung function^2,3^ compared to conventional ventilation therapy. However, three decades after the introduction of HFOV^4^, the underlying mechanisms of gas exchange during this technique have still not been fully understood^4,5^. Previously, eight potential mechanisms of gas exchange were suggested explaining oxygen delivery to the terminal lung regions even for tidal volumes much smaller than respiratory dead space^6^. Due to ethical limitations on invasive measurements in vulnerable preterm infants, none of these mechanisms has been measured clinically and their existence remains an elaborate guess to date.

Recently, computational methods have been used to simulate air flow and gas transport during HFOV and to elucidate the efficiency of HFOV in oxygen and CO_2_ transport within the airways^7–9^. While providing important first insights, the computational methods have not been available for investigations on HFOV in the preterm infant. Herrmann *et al*^9^. investigated gas transport during HFOV in the canine lung while the work in^7,8^ was limited to artificial or imaging-based adult airway tree geometries. Further, none of the previously reported approaches was able to respect the mechanics of the respiratory system of the infant and the effect of tube leakage, which is common in neonatal ventilation.

The computational lung model presented in this work was designed to overcome the aforementioned limitations. The used bronchial anatomy is derived from highly resolved Magnetic Resonance Imaging (MRI) of a preterm infant and comprises the first seven generations of the bronchial tree as well as the used non-blocked endotracheal tube. Infant Lung Function Testing (ILFT) results are included to respect lung mechanics of the patient, which governs the passive behaviour of the lung especially during expiration. This novel computational lung model can be subjected to representative conditions of HFOV and is perfectly suited to reconfirm the previously hypothesised gas transport mechanisms in HFOV^6^.

## Results

### Equilibrium state conditions in the model under HFOV

**Figure 1:**
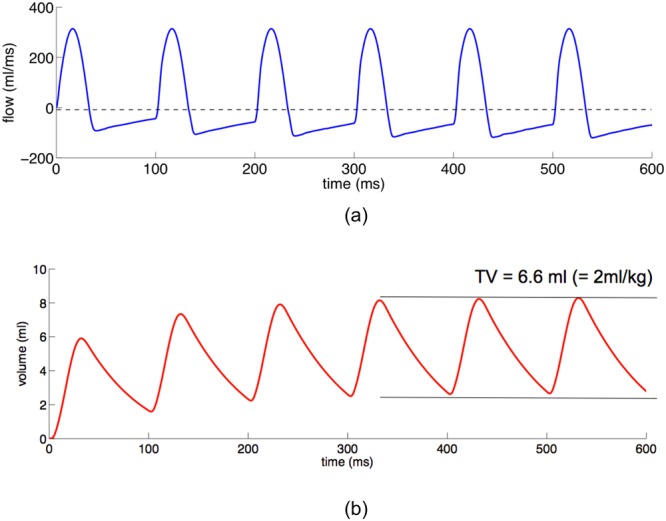
(**a**) Shows the monitored airflow at the proximal end of the endotracheal tube over six breathing cycles. During the inflow phase, the prescribed sinusoidal flow is correctly represented. During the outflow phase flow is determined by the mechanics of the lung. (**b**) Shows the volume curve over time. Lung volume increases during the first four breaths (t ≤ 400 ms) and remains stationary afterwards indicated by the two horizontal lines in the graph.

To assure clinically realistic airflow conditions in the HFOV model global flow monitoring is conducted at the proximal end of the endotracheal tube and visualised in Fig. 1a over six breathing cycles. During the inspiration phase (e.g., t < 33 ms) flow is sinusoidal as prescribed by the HFOV settings. During exhalation (e.g., 33 ms < t < 100 ms) the flow develops a tidal expiratory flow pattern with a small outflow detected before the next forced inflation starts (see e.g., t = 100 ms). The tidal volume curve, visualised in Fig. 1b indicates that during the first three inflations, absolute lung volume still increases and that after the fourth inspiration, a steady state between the mechanics of the respiratory system and the ventilator in HFOV mode is achieved. An observed tube leakage of approximately 16% of the supplied tidal volume is in line with clinical findings^10^.

### Gas exchange mechanisms

**Figure 2:**
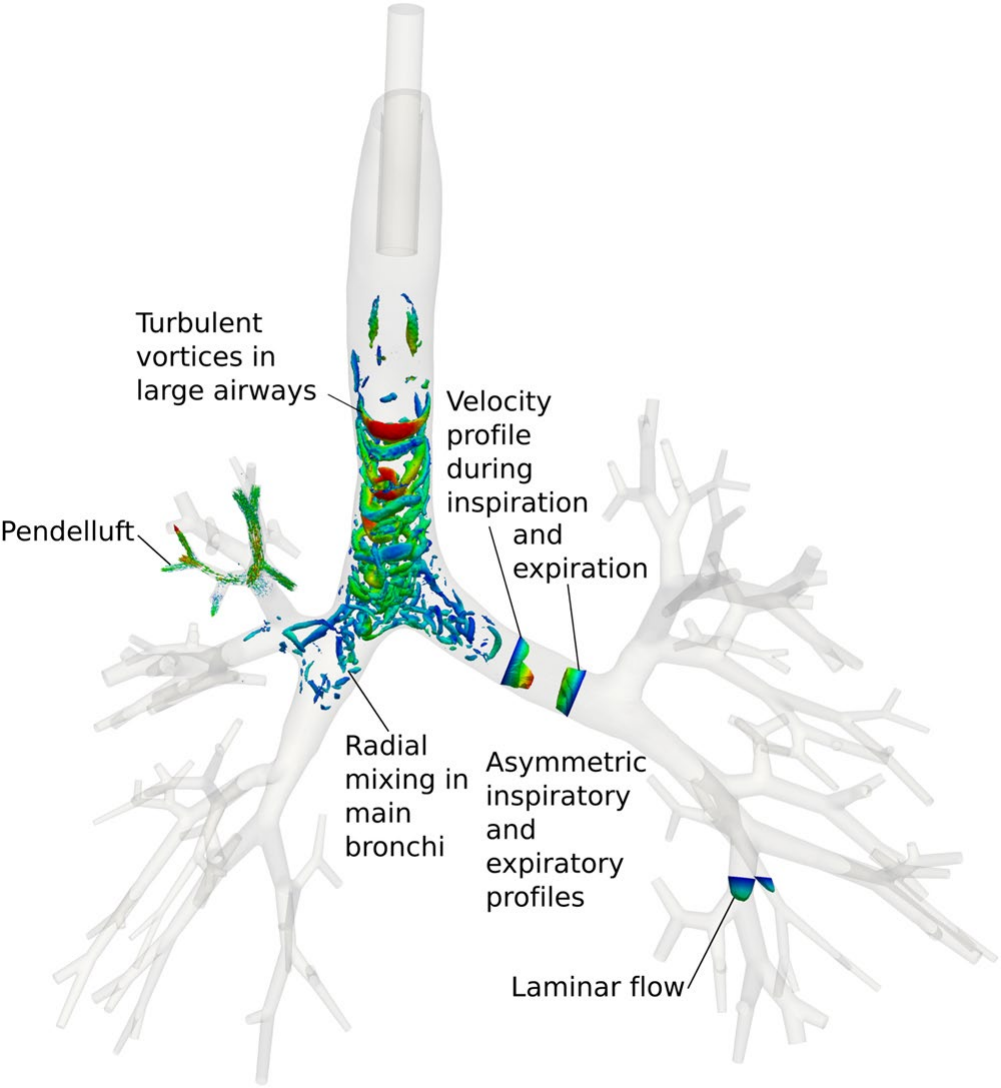
Gas exchange mechanisms detected by the computational lung model and their location in the anatomy of the preterm infant. To illustrate the emerging turbulence during the inspiration phase, λ2 iso-surfaces as a widely used criterion for turbulence are visualised in the larger airways in Fig. 2.

Observing the computed airflow at a steady state after the fourth cycle at high spatial and temporal resolution, six particular flow and gas transport mechanisms are observed and visualised in Fig. 2.

#### Turbulent vortices in large airways

One major aspect that is non-intuitive in HFOV is the fact that oxygen can be transferred towards the respiratory units of the lung although tidal volume is much smaller than respiratory dead space. Here, turbulence plays the decisive role in transporting oxygen rapidly through the large airways. Λ_2_ iso-surfaces as a widely used criterion for turbulence are visualised in Fig. 2 illustrating the development of a vortex during the inspiratory phase that allows oxygen to travel faster on its surface than the average flow velocity of air. By this vortex, the dead space of the major airways and especially of the trachea is easily overcome. During the expiratory phase, no turbulence is observed in the large airways. Temporal evolution of turbulence in large airways is provided in the supplementary video S4.

#### Asymmetric inspiratory/expiratory profiles

A further mechanism of efficient gas exchange during HFOV is the asymmetry between inspiratory and expiratory flow profiles. During the inspiration phase, the velocity profile shows elevated velocity values for oxygen-rich gas in the lower part of the airway cross-section (Fig. 2). During the expiratory phase, the velocity profile indicates higher values in the upper part of the cross-section. Due to this asymmetry, oxygen-rich gas is transported faster in the lower part of the airway cross-section and low-oxygen gas is efficiently cleared in the upper part of the cross-section.

#### Radial mixing in main bronchi

Turbulent flow and radial mixing promotes efficient gas exchange in HFOV. A prominent location for this is the right main bronchus. As visualised in Fig. 2 and in the supplementary video S4, the emerging turbulence introduces a secondary “stirring” motion into the fluid flow. This flow pattern leads to an efficient radial mixing of oxygen within the gas inflated into the lungs. During the expiratory phase this radial mixing decays and ensures that CO_2_ is removed efficiently from the right main bronchus.

#### Laminar flow in higher generations

Laminar flow is observed in higher generations of the bronchial tree, mainly in those airways at a generation number higher than six. Downstream of these regions, airflow is decelerated for optimal diffusive transport of the gas into the distal lung region.

#### Pendelluft

**Figure 3:**
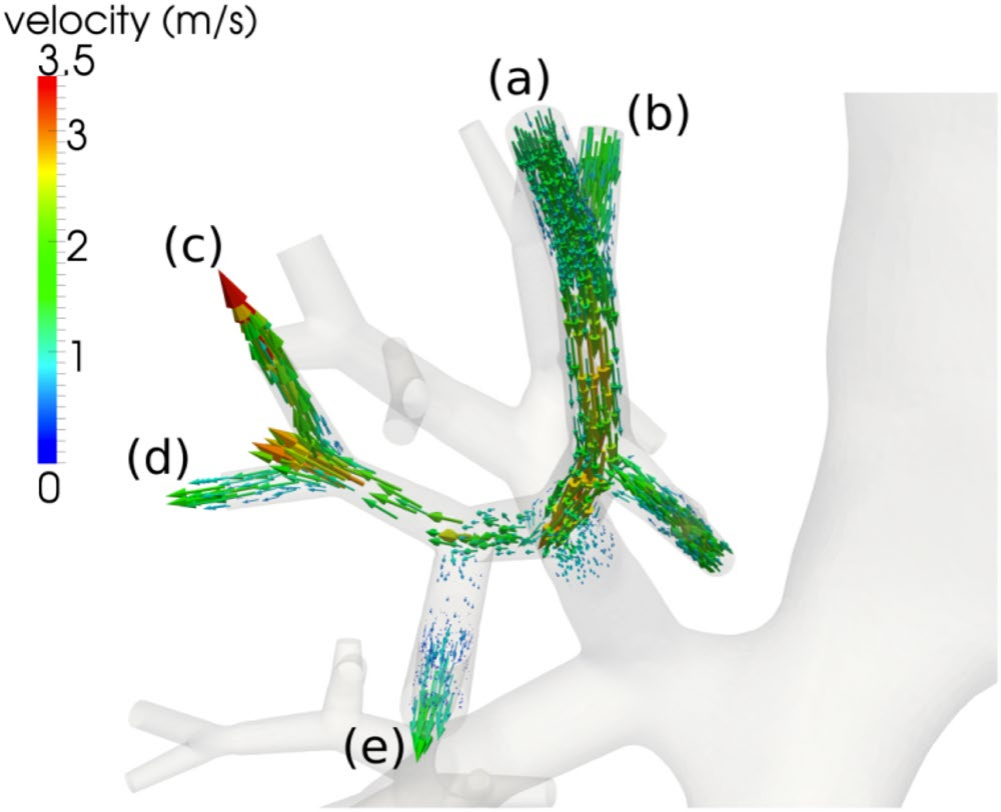
Pendelluft flow occurring at five airway branches in the bronchial tree of the investigated preterm infant shortly before the end of the inspiration phase (t = 32 ms). The arrows represent airflow velocity and indicate a flow from the regions (**a**,**b**) into the regions (**c**–**e**).

Different compliances between healthy lung segments and those affected by BPD in our preterm infant can lead to regional differences in dynamic pressure and induce an airflow between these regions known as pendelluft^11^. The different regional compliances of lung tissue, e.g., in the right upper lobe of our patient, lead to an airflow from regions (a) and (b) to the regions (c), (d), and (e) just before just before the end of the inspiration phase at t = 32 ms (see Fig. 3).

#### Direct ventilation of central alveoli

**Figure 4:**
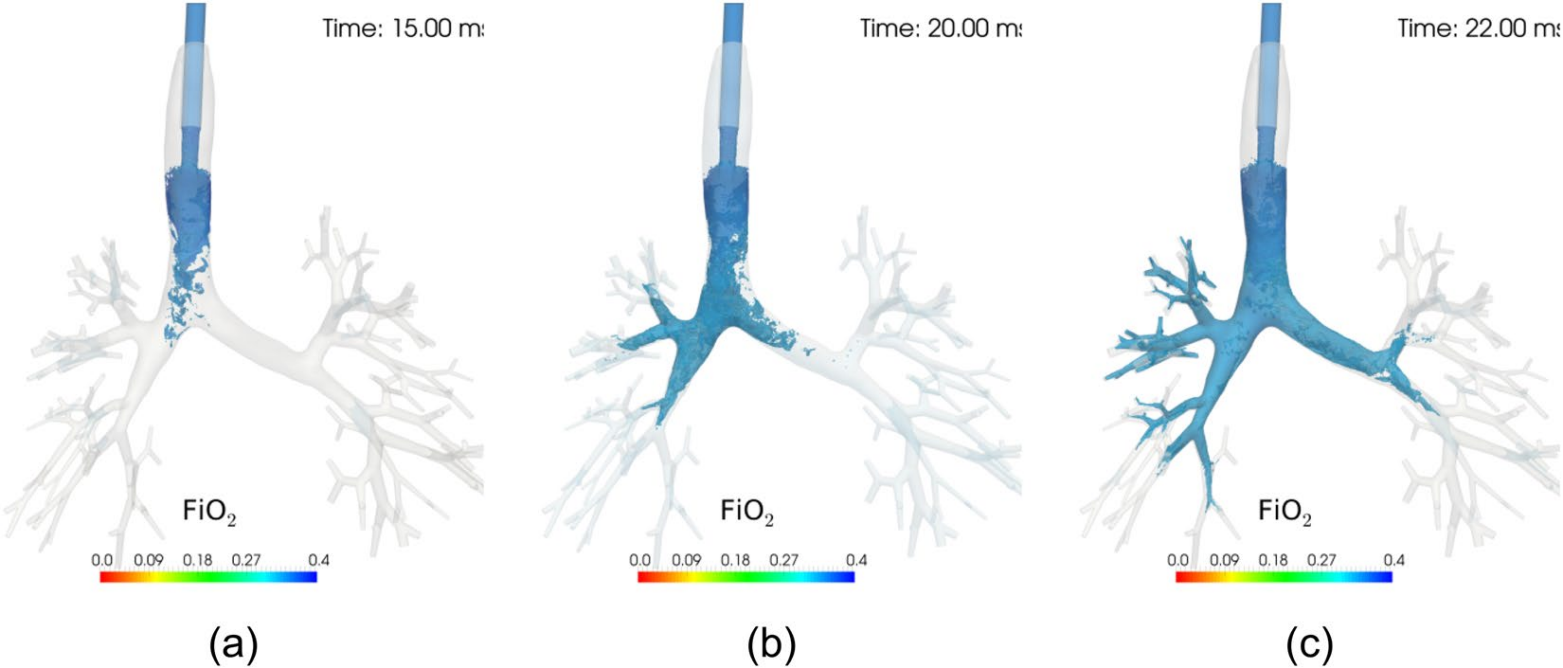
Temporal evolution of oxygen transport during a single HFOV cycle. The visualisation refers to the fourth ventilation cycle and annotated time starts at the beginning of the fourth cycle. The region marked in blue shows the fraction of inspired oxygen larger than 0.25 and is coloured according to the visualised scale. The distribution shows that regions close to the major airways (e.g., the right upper lobe) receive oxygen directly during inflation while others such as the left inferior lobe receive oxygen only within the next ventilation cycle with oxygen partly delivered throughout the tree in the current inflation. The effects of turbulence on oxygen transport can be clearly seen in (**a**) and of asymmetry in the inspiratory velocity profile in the left main bronchus in (**b**).

Figure 4 shows the stream of oxygenised air delivered into the lungs at three points in time. Lung regions located close to the major airways become directly ventilated, especially in the right upper lobe, while other areas of the lung do not have direct contact with oxygen during the first inflation. In these areas oxygen is transported further downwards into the distal airways during the subsequent cycles of ventilation. For a visualisation of the temporal evolution of oxygen transport throughout a full cycle the reader is referred to supplementary video S5.

## Discussion

### Additional insight into pulmonary physiology

This study confirms the existence and shows the exact location of gas transport mechanisms in the preterm infant under HFOV (*f* = 10 Hz, *V*_*T*_ = 2.0 ml kg^−1^) previously suggested by Slutsky and Drazen^6^. Based on our data, knowledge on HFOV is no longer limited to global measurements on consumed oxygen but supported by the underlying physics of regional airflow and gas transport in the infantile lung. In conclusion, six gas transport mechanisms were observed during HFOV. Each mechanism has been observed at a specific location in the bronchial tree and at a specific time during each breath to optimally supply oxygen to the respiratory region and to efficiently clear CO_2_ from the infantile lung.

Further work will be performed to clarify whether certain mechanisms can be triggered by special HFOV parameter settings e.g., proposed by^12,13^. With the described model in hands we are now capable of modifying ventilator settings for a specific clinical situation in silico and may even predict the effect of these setting-changes on both oxygenation and CO_2_-elimination supporting interesting clinical studies such as^12,13^.

### Limitations

There are limitations to our study that need to be taken into account when interpreting presented data. First, the medical imaging data used in this study were obtained from a preterm infant at the age of 3 months and might underestimate the flow situation in an extremely preterm (and even smaller) neonate that is subjected to HFOV early during the clinical course. Usually MRI scans in a 3 T scanner with the applied protocol are not available for a neonate in the first weeks in a standard clinical setting. Therefore, one future goal in bringing the presented methods into clinical practice is to use clinical standard radiographic (X-ray) images as basis for lung model generation.

Also, oxygen is assumed to be consumed if it leaves the outlet after the 7th generation of the bronchial tree. This aspect limits the model to a conservative estimation of real gas exchange, as oxygen would be partly stored in the lower airways and could re-enter the domain in the next breath for mixture with further oxygen-rich air.

This work is limited to the observation of convective and convective-diffusive mechanisms in the first eight generations of the bronchial tree, which comprises six out of eight phenomena proposed in the article by Slutsky and Drazen^6^. The remaining two diffusive mechanisms might be detectable in combination with detailed models of the respiratory zone. Hofemeier *et al*.^14^ have e.g., provided first steps towards tracking diffusion processes in lung tissue, which can be adopted to study the remaining diffusion and collateral ventilation on the alveolar scale.

Finally, a clinical observation or validation of the proposed mechanisms is extremely difficult and has not been possible up to now. One especially promising method for such a validation in the same geometry that has been used for the computations would be hyperpolarised ^3^He MRI imaging following previous ideas^15,16^. Another method for experimental validation is 3D printing and ventilating a rigid full-scale model of the investigated infantile bronchial tree in this study with all limitations outlined in^17^, especially the lack of realistic outflow boundary conditions to store the air during inspiration and to create a backflow in the expiratory phase.

## Conclusion

In this work, we provide evidence for six mechanisms of gas exchange during high-frequency oscillatory ventilation (HFOV) in preterm infants, namely: (i) turbulent vortices in large airways; (ii) asymmetric inspiratory/expiratory velocity profiles; (iii) radial mixing in main bronchi; (iv) laminar flow in higher generations of the respiratory tract; (v) pendelluft and (vi) direct ventilation of proximal regions of the lung.

Not only confirming their existence but also showing the exact location of gas transport mechanisms in the preterm infant under HFOV gives additional insight into pulmonary physiology. Our in silico model has the potential to enable clinicians to predict the effect of ventilator setting-changes on gas exchange, both oxygenation and ventilation, in an individual patient. Thus, in the future, potentially harmful try and error adaptations of ventilator settings might be prevented.

Limitations on measurements and sequential medical imaging, especially in the preterm infant are overcome by using an advanced computational lung model based on the real anatomy and physiology of the infantile lung. This detailed insight into HFOV promotes a better understanding of this successful ventilation technique in theory and provides a further step towards optimal ventilatory assistance to the immature lung.

## Material and Methods

### Patient characteristics

A preterm infant from the AIRR study cohort (Attention to Infants @ Respiratory Risk) with later development of Bronchopulmonary Dysplasia (BPD) born at 27 3/7 weeks of gestation at the Perinatal Center of the University Hospital, LMU-Munich, Campus Grosshadern was chosen for all investigations in this work. Patient characteristics are described in Table 1. Approval by the local Ethics Committee (Ethical Committee of the Medical Faculty, LMU-Munich) and written informed parental consent for the study infant was obtained (Munich cohort #195-07; German Registry for Clinical Studies DRKS00004600).

**Table 1:** Patient characteristics of the preterm infant investigated in this work.

BPD grade	3
Gestational age (weeks PMA)	27.3
Birth weight (g)	760
Days of mechanical ventilation	78
Endotracheal mechanical ventilation (n/days)	32
Pharyngeal ventilation/CPAP (n/days)	46
ICU stay (days)	103

BPD, Bronchopulmonary Dysplasia, graded according to the definition in^29^; PMA, post-menstrual age; CPAP, continuous positive airway pressure; ICU, intensive care unit.

Infant Lung Function Testing (ILFT) was performed at 36 weeks GA under light sedation with chloralhydrate (30 mg/kg). Measurements were standardized according to the recommendations of the American Thoracic Society (ATS) and European Respiratory Society (ERS)^18,19^. Measurements of tidal breathing, passive respiratory mechanics and functional residual capacity (FRC_p_) were performed. Total respiratory compliance (C_rs_) was assessed in single occlusion technique (SOT) using five to eight regular tidal breaths to establish a stable end-expiratory level (EEL) before activating the balloon shutter. FRC_p_ was measured in the bodyplethysmograph as described previously with the infant making respiratory efforts against a closed shutter not using the raised volume technique^20,21^.

Following previous recommendations^21^ a minimum of five technically satisfactory manoeuvres were used to calculate the mean C_rs_ and FRC_p_ from 3–5 valid measurements. For our patient a C_rs_ of 20.93 ml kPa^−1^ was determined and used in lung model calculation.

### Pulmonary MRI at 36 weeks GA

Pulmonary native MRI measurements were performed in non-sedated, spontaneously breathing infants in supine position in a 3-Tesla whole-body MRI scanner (Magnetom Skyra, Siemens Healthineers, Erlangen, Germany). Details of the MRI protocol are given in the supplementary material S1. For lung geometry extraction in this study, a T1-weighted magnetization-prepared rapid gradient-echo (MP-RAGE) sequence with an image size of 256 × 256 × 144 px^3^ was chosen.

### Lung model generation

**Figure 5:**
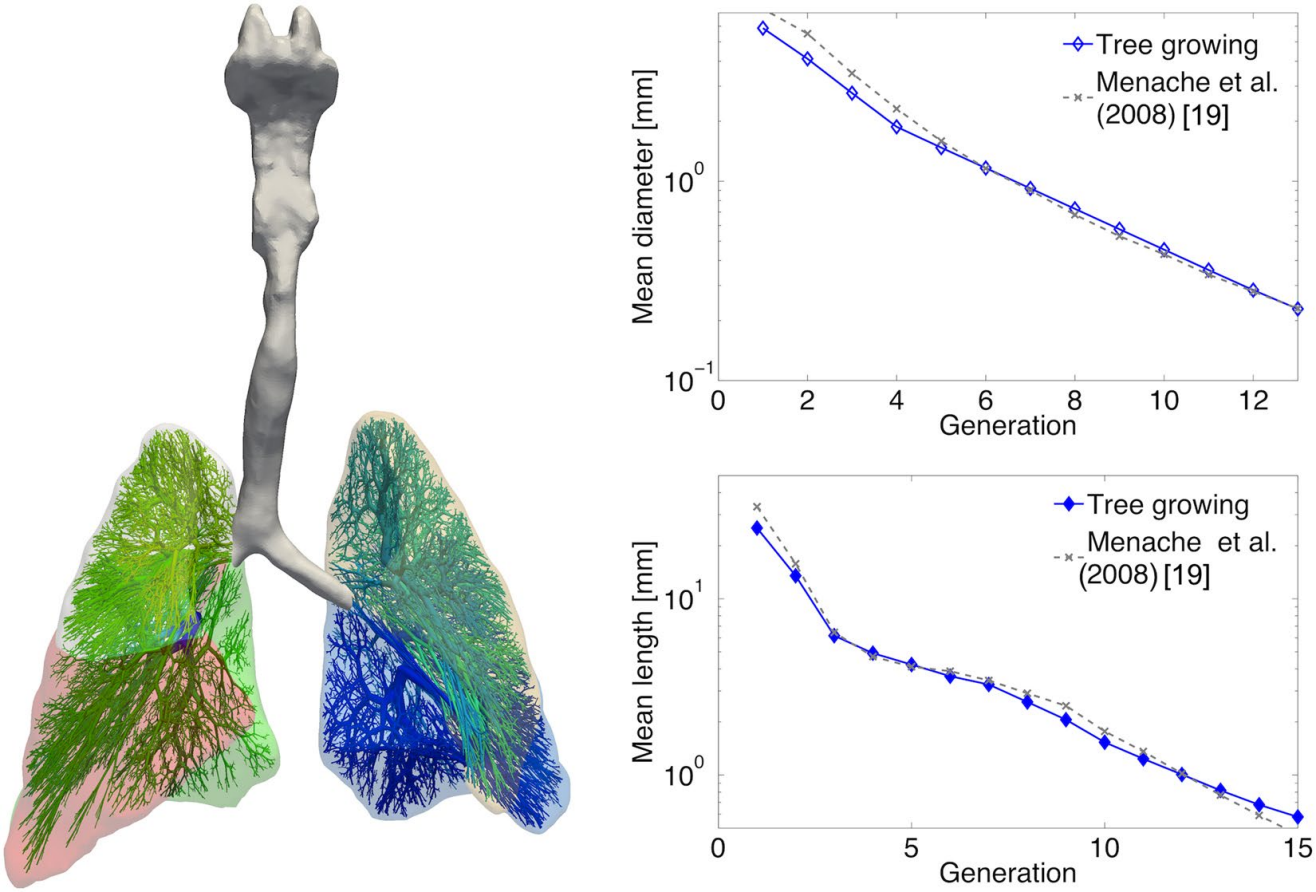
(left) shows the reconstructed upper airways, parts of the lower airways and the hull geometry of the left and right lung. Different colours refer to the individual lung lobes of the preterm infant. The resulting mean lengths and diameters of the model airway tree correspond well to previously reported measurements as indicated in (right).

The first seven generations of the bronchial tree of the preterm infant were modelled as a fully resolved three-dimensional computational domain reconstructed from the MR images following the model setup outlined in the supplementary material S2. The airway tree morphometry is shown in Fig. 5 and corresponds well to previously reported values for preterm infants^22^. The entire airway tree had a respiratory dead space of 2.2 ml kg^−1^ body weight corresponding to previously reported clinical measurements of 2.51 ± 0.61 ml kg^−1^ ^23^.

**Figure 6:**
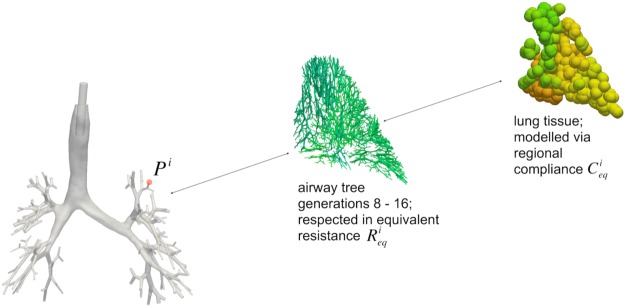
Fully resolved bronchial tree of the first seven airway generations (left). Starting at one exemplary outlet (marked in red), the associated further downstream generations 8–16 are visualised in green (mid). The resistance of these airways marked in green is respected via their equivalent resistance R_eq_^i^. The compliance C_eq_^i^ of the lung region corresponding to the exemplary (red) outlet is obtained from ILFT.

Conducting airways located downstream to generation seven are respected by their equivalent resistance ${{\rm{R}}}_{{\rm{eq}}}^{{\rm{i}}}$ (see Fig. 6). Similarly, lung mechanics of each region corresponding to a subdivision after generation seven is represented by a regional compliance ${{\rm{C}}}_{{\rm{eq}}}^{{\rm{i}}}$ computed from the ILFT. The pressure P^i^ at the end of generation seven then reads

1 $${{\rm{P}}}^{{\rm{i}}}={{\rm{R}}}_{{\rm{eq}}}^{{\rm{i}}}\ast {{\rm{Q}}}^{{\rm{i}}}+{{\rm{\Delta }}V}^{{\rm{i}}}/{{\rm{C}}}_{{\rm{eq}}}^{{\rm{i}}}$$

where the superscript i denotes the single regions and the volume change ∆V^i^ results from integration of the flow Q^i^ into this region over time. Using this formulation, (i) the correct dynamic pressure at the end of the fully resolved tree can be computed and (ii) the volume of air entering the single tissue regions can be stored for consistent modelling of expiration based on the mechanics of the lung.

At the upper end of the trachea an un-blocked endotracheal tube with an inner diameter of 3.0 mm is placed according to clinical guidelines. The remaining cross-sectional area of the leak between the pharynx and the tube is measured from the imaging data and included in the model to allow for tube leakage in the preterm infant.

### HFOV settings

For the in-silico simulation a PEEP of 8 cm H_2_O was chosen to be constantly applied at the proximal end of the endotracheal tube. Further, a sinusoidal flow rate was superimposed during the inflow phase with a frequency of *f* = 10 Hz, a tidal volume of *V*_*T*_ = 2.0 ml kg^−1^ body weight of the preterm infant and an inspiratory/expiratory ratio of I:E = 1:2. Expiratory flow was allowed to freely develop depending on regional lung mechanics, i.e., R_eq_^i^ and C_eq_^i^, and the applied PEEP. A mean volume fraction of inspired oxygen of 35% (FiO_2_ = 0.35) was used.

### Governing equations for fluid and transport dynamics

Airflow in the fully resolved three-dimensional bronchial tree is governed by the incompressible Navier-Stokes equations (for details on the formulation see supplementary material S2). In good approximation, air is seen as Newtonian fluid with a kinematic viscosity of ν = 17 mm^2^ s^−1^ and a density of ρ = 1.27 kg m^−3^.

Gas transport in the model is governed by the convection-diffusion equation for the concentration of oxygen Φ. The equation reads

2 $$\partial {\rm{\Phi }}/\partial {\rm{t}}+{\bf{u}}\ast \nabla {\rm{\Phi }}-\nabla ({\rm{D}}\nabla {\rm{\Phi }})=0$$

where **u** denotes the airflow velocity resulting from the solution of the incompressible Navier-Stokes equations and D the diffusion coefficient for oxygen in air (D = 0.219 cm^2^ s^−1^). The boundary conditions for the oxygen concentration at the tube inlet are

$${\rm{\Phi }}=0.35$$

and

$$\partial {\rm{\Phi }}/\partial {\bf{n}}=0$$

with **n** being the normal vector at the outlets of the first seven airway generations of the bronchial tree.

Fluid dynamics and scalar transport equations are solved via stabilised finite elements^24^ in our in-house code, which has been used successfully in several biomedical flow and transport applications (see e.g.^25–28^). For validation of the presented computational methods in the course of HFOV, an available benchmark test setup is simulated and results are verified against available literature data in the supplementary material S3.

## Electronic supplementary material

Supplementary Material

Temporal evolution of airflow patterns in the preterm infant

Temporal evolution oxygen concentration in the preterm infant
