# Supplementary material for: Gas exchange mechanisms in preterm infants on HFOV – a computational approach
Source: Sci Rep. 2018 Aug 29;8:13008. doi: 10.1038/s41598-018-30830-x (PMC6115430; doi:10.1038/s41598-018-30830-x)
Supplement: Supplementary file 1 — Supplementary Material [file 41598_2018_30830_MOESM1_ESM.docx]

Gas exchange mechanisms in preterm infants on HFOV – a computational approach: Supplementary Material

Christian J. Roth^1,a^, Kai M. Förster^2,3,a^, Anne Hilgendorff^2,3^, Birgit Ertl-Wagner^4^, Wolfgang A. Wall^1,b^ and Andreas W. Flemmer^2,b,c^

^1^ Institute for Computational Mechanics, Technical University of Munich, 85748 Garching, Germany

^2^ Division of Neonatology, Dr. von Hauner Children's Hospital, Perinatal Center Grosshadern, Ludwig-Maximilian-University, 81337 Munich, Germany

^3^ Comprehensive Pneumology Center, Helmholtz Zentrum München, Munich, Germany, Member of the German Lung Research Center (DZL)

^4^ Institute for Clinical Radiology, Ludwig-Maximilian-University, 81377 Munich, Germany

___________________________________________________________________

a Both authors contributed equally as first authors

b Both authors contributed equally as senior authors

c Corresponding author: Division of Neonatology, Dr. von Hauner Children's Hospital, Perinatal Center Grosshadern, Ludwig-Maximilian-University, Marchioninistrasse 15, 81337 Munich, Germany, phone: +49-89-4400-72800/01, email: Andreas.Flemmer@med.uni-muenchen.de

**S1. Details on the MRI protocol**

Magnetic Resonance Imaging was performed with a size-adapted number of coil elements from the 32-channel spine array coil, an 18-channel flexible body array coil and the 20-channel head-and-neck array coil. The protocol included pulse sequences for the qualitative and quantitative assessment of morphology, volume, and structural changes of the lung. In detail, the following pulse sequence was used: T2-weighted single-shot fast-spin-echo (ssFSE) sequences in coronal, axial, and sagittal orientation; spatial resolution 1.9×1.3×4.0 mm³, 20 slices with a field of view (FOV) of 340×255 mm². The echo time (TE) was 57 ms; the acquisitions were ECG-triggered with a minimum repetition time (TR) of 2 RR intervals; 2 signal averages were acquired. The total acquisition time of the three T2-weighted ssFSE sequences was about 5 minutes.

**S2. Lung model generation steps**

The upper airways, the first generations of the lower airways and the contours of single lung lobes were directly segmented from the MR images using the software package Mimics (Materialise, Leuven, Belgium). Further, an un-blocked endotracheal tube with an inner diameter of 3.0 mm was placed in the trachea according to clinical guidelines.

To include further generations of the bronchial tree beyond those that are directly segmentable from the MR images, a recursive tree growing algorithm proposed for human adults [1,2] was adopted to mimic preterm infant anatomy. Tree growth started at the last fully segmentable airways and the next airway generation bifurcates relative to the direction given by the last segmentable airway. A tree was then grown by recursively splitting a parent airway into two daughters according to morphological length and diameter ratios reported previously [3,4]. Tree growth was terminated if a minimal airway length of l_min_ = 0.36 mm, or a minimal airway diameter of d_min_ = 0.049 mm was reached, if the maximum generation number of n_max_ = 17 was exceeded, or if an airway touched the segmented hull geometry of the lobes.

The resulting airway tree morphometry is shown in Fig. 1 and corresponds well to previously reported values for preterm infants at this age [3]. Also, the total number of 24,880 generated terminal airways supplying the pulmonary acini is in line with reported values in literature [5,6]. The entire airway tree had a respiratory dead space of 2.2 ml kg^-1^ body weight corresponding to previously reported clinical measurements of 2.51±0.61 ml kg^-1^ [7].

The first seven generations including the endotracheal tube were modelled as a fully resolved three-dimensional domain with 128 outlets (see Fig. 2). The geometry was meshed with a resolution of 0.01 mm using Gmsh (version 2.9.3; [8]) resulting in 5,109,584 tetrahedral elements. This is similar to the resolution provided in [9] and approximately three times higher than in [10].

All airways located downstream to one of the 128 outlets in generation 8-16 were combined to an equivalent resistance $R_{eq}^{i}$ that is computed from the arrangement of the single airways within the tree and their individual airway resistance $R^{j}$. Individual airway resistance $R^{j}$ is based on the model by van Ertbruggen et *al.* [11] and reads

$R^{j}=\left\{\begin{matrix} \sqrt{\gamma\cdot\mathrm{Re}\cdot D/L}\cdot R_{p}\text{ if }\mathrm{Re}\geq L/(\gamma^{2}\cdot D) \\ R_{p}\text{ if }\mathrm{Re}\geq L/(\gamma^{2}\cdot D) \end{matrix}\right.$ (S1)

with the Poiseuille resistance

$R_{p}=\frac{8\cdot\nu\cdot\rho\cdot L}{\pi\cdot(D/2)^{4}}$ (S2)

and the Reynolds number $\mathrm{Re}$ defined as

$\mathrm{Re}=\frac{u\cdot D}{\nu}$. (S3)

The variable $D$ denotes the diameter and $L$ the length of the airway, and the kinematic viscosity $\nu=17\text{ mm}^{2}\text{ s}^{\text{-1}}$, the density of air $\rho=1.27\text{ kg m}^{\text{-3}}$ and the velocity $u$are physical flow quantities. The generation-dependent prefactor $\gamma$ is taken from [11] and given in Table S1. We might annotate that the resistance model in [11] is only valid during inspiration and generally airway compliance would have to be considered. However, during HFOV, the pressure variations are small enough that no significant changes of the airway diameter occur and the model reported in [11] remains valid for both inspiration and expiration.

| **Table S1**. Values for the generation-dependent resistance prefactor $\gamma$ taken from [11] |
| --- |
| | Generation | 0 | 1 | 2 | 3 | 4 | 5 | 6 | 7 | >7 | | --- | --- | --- | --- | --- | --- | --- | --- | --- | --- | | $\gamma$ | 0.162 | 0.239 | 0.244 | 0.295 | 0.175 | 0.303 | 0.356 | 0.566 | 0.327 | |

Similar to the resistance of an individual airway, an equivalent resistance of the upper airways with the placed endotracheal tube is computed. Thereto, the cross-sectional area of the leak between the pharynx and the tube is extracted from the imaging data. The resulting resistance $R_{eq}^{tl}=\text{100 cmH}_{2}\text{O l}^{\text{-1}}\text{ s}$ is attached to the three-dimensional flow model at the upper end of the trachea to respect tube leakage in the preterm infant.

Each of the 128 tree outlets defined above supplies one lung region with an approximate diameter of 10 mm. A regional compliance $C_{eq}^{i}$ is computed to represent the mechanics of each lung region. Regional compliance is assumed to be constant due to (i) sufficiently small pressure variations in HFOV (in our case approximately ±2.5 cmH_2_O), and (ii) the PEEP which ensures that the lung is kept within the linear part of the pressure-volume curve. Following the derivation by Ma and Bates [12] the measured total compliance of the respiratory system C_rs_ (see Section 2.1) is equally distributed to

$C_{eq}^{*}=\frac{1}{N}C_{rs}$ (S4)

where N denotes the number of lung regions, i.e., in our case N = 128. To additionally account for the variation in regional compliance caused by the BPD, a prefactor $c$ is introduced depending on the average pixel gray value of each tissue region in the MR image. Gray values in the MR image of the preterm infant’s lung range from 0 to 695. This variation is transferred towards a variation of regional compliances of 0.13 to 0.26 ml kPa^-1^ (i.e., 30% variation from the mean value $C_{eq}^{*}=0.164\text{ ml kPa}^{\text{-1}}$) as visualised in Fig. S1.

| 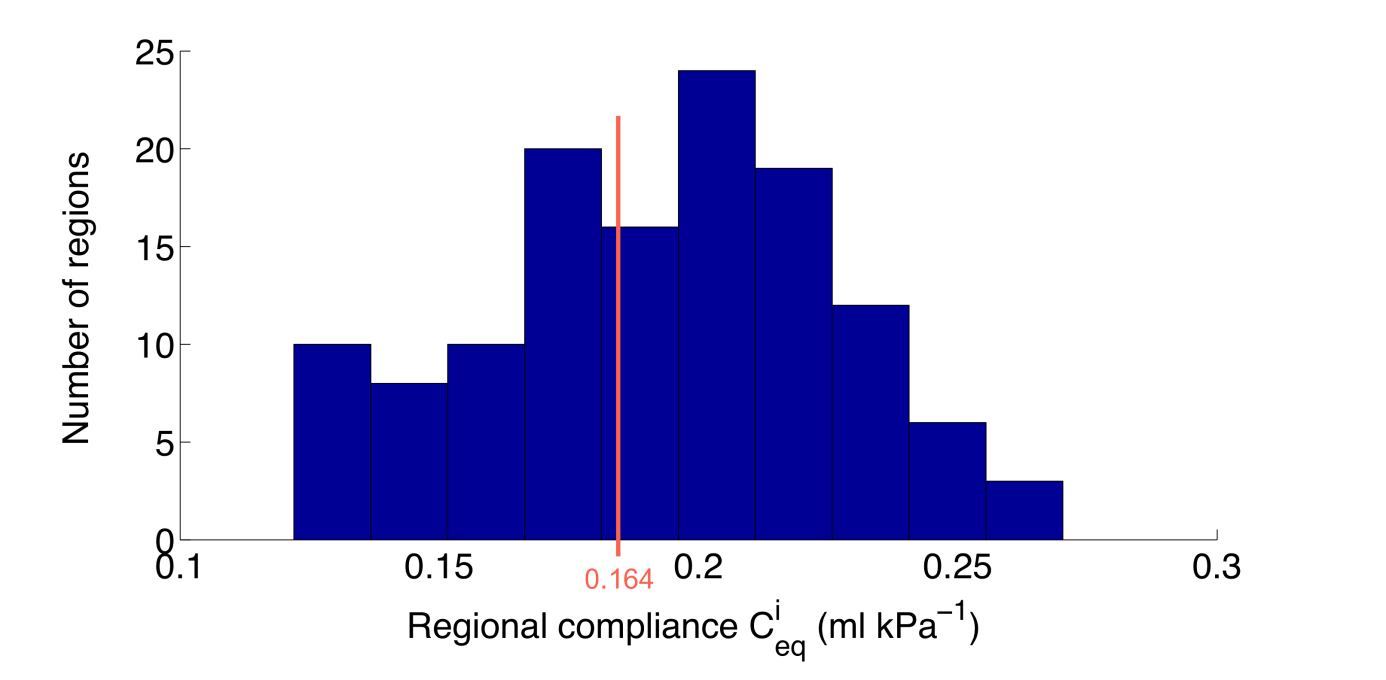 |
| --- |
| **Fig. S1**. Histogram distribution of the regional compliance values used to mimic the behaviour of lung tissue in this study. The red line indicates the average regional compliance value obtained from the formula by Ma & Bates [12]. |

With this prefactor, Eq. (S4) reads

$C_{eq}^{i}=c\cdot C_{eq}^{*}$ (S5)

and the regional compliance can be computed. Finally, at each of the 128 outlets of the fully resolved bronchial tree the regional resistance and compliance of the further downstream regions are respected via Eq. (1).

Airflow in the fully resolved three-dimensional bronchial tree is governed by the incompressible Navier-Stokes equations reading

$\begin{matrix} \frac{\partial\mathbf{u}}{\partial t}+\left(\mathbf{u}\cdot\nabla\mathbf{u}\right)+\nabla p-2\nu\nabla\mathbf{u}\cdot\varepsilon\left(\mathbf{u}\right)=\mathbf{f} \\ \nabla\cdot\mathbf{u}=\mathbf{0}. \end{matrix}$ (S6)

Here, $\mathbf{u}=\mathbf{u}\left(\mathbf{x},t\right)$ denotes the airflow velocity and $p=p\left(\mathbf{x},t\right)$ the kinematic pressure with respect to spatial coordinates $\mathbf{x}=\left(x_{1},x_{2},x_{3}\right)^{T}$ and time $t$. The variable $\nu$ represents the kinematic viscosity of air, and the vector $\mathbf{f}$ stands for given body forces on the fluid. The deformation rate tensor of the fluid is defined as

$\varepsilon\left(\mathbf{u}\right)=\frac{1}{2}\left(\nabla\mathbf{u}+\left(\nabla\mathbf{u}\right)^{T}\right).$ (S7)

The boundary conditions for fluid flow read

$$\left\{\begin{matrix} Q(t)=\frac{d}{dt}\left(0.5\cdot6.6\text{ }ml\cdot\left(1-\cos\left(\frac{\pi\cdot t}{33\text{ ms}}\right)\right)\right);\text{ t}\in\text{t}_{\text{exp}} \\ p(t)=PEEP;\text{ t}\in\text{t}_{\text{exp}} \end{matrix}\right.$$

with $\left(n\cdot100\right)\text{ ms}\leq t_{in}\leq\left(n\cdot100+33\right)\text{ ms}$ denoting the inspiratory phase and $\left(n\cdot100+33\right)\text{ ms}\leq t_{\exp}\leq\left((n+1)\cdot100\right)\text{ ms}$ the expiratory phase.

Gas transport in the model is governed by the convection-diffusion equation for a scalar concentration field $\Phi$ - in our case the concentration of oxygen. The equation reads

$\frac{\partial\Phi}{\partial t}+\mathbf{u}\cdot\nabla\Phi-\nabla\left(D\nabla\Phi\right)=0$ (S8)

where $\mathbf{u}$ denotes the airflow velocity resulting from the solution of Eq. (S6) and $D$ the diffusion coefficient for oxygen in air ($D=0.219\text{ cm}^{2}\text{ s}^{\text{-1}}$). The boundary conditions for the oxygen concentration at the tube inlet are $\Phi=0.35$ and $\frac{\partial\Phi}{\partial n}=0$ with n being the normal vector at the outlets of the first seven airway generations of the bronchial tree.

Airflow velocity and scalar transport are one-way coupled which means that the flow solution influences the solution of scalar transport but not vice versa, which is reasonable in the present case of convective-diffusive scalar transport [13]. It is further important that all Neumann boundary terms are included in the weak finite element formulation of the fluid dynamics equations (Eq. (S6)) following the formulation in [14]. Otherwise a mathematical incomplete formulation would introduce flow instabilities during the expiration phase when no (unphysiological) flow velocity is prescribed at the inlet and expiratory flow is rather driven by the elastic recoil of the lung.

The simulation is run for six ventilator cycles (i.e., 600 ms) with a temporal resolution of 0.01ms to highly resolve all turbulence phenomena in space and time. Convergence of the fluid dynamical problem is achieved if the non-linear residual in each time step is smaller than 10^-6^.

**S3. Lung model validation**

For validation of the presented computational methods, an available experimental benchmark test is set up and simulations are verified against available literature data. Following the example by Choi et *al.* [9] a straight three-dimensional tube with a radius of $a=4.6\text{ }mm$ and a length of $10a$ is created. The tube is meshed with hexahedral elements specifying a spatial resolution of 0.30 mm.

In order to set up a case where an analytical solution is available, oscillatory flow with a single frequency is simulated. Therefore, the inlet of the tube (i.e., the left cross-sectional area) is subjected to an oscillatory inflow velocity $u_{a}$ in axial direction according to the analytical Womersley solution reading

$u_{a}\left(r,t\right)=Re\left\{\frac{U_{0}}{\rho}\frac{1}{i\omega}\left(1-\frac{J_{0}\left(\alpha\cdot r/a\cdot i^{3/2}\right)}{J_{0}\left(\alpha\cdot i^{3/2}\right)}\right)e^{i\omega t}\right\}$. (S9)

Here, $U_{0}$ denotes the amplitude and $\omega$ the angular frequency of the harmonic velocity oscillation. The variable $\alpha$ denotes the Womersley number defined as $\alpha=a\sqrt{\omega/\nu}$, $r$ the radial coordinate in the tube with $-a\leq r\leq a$, $i$ the imaginary unit and $J_{0}$ the 0-th Bessel function of the first kind. Being a physical quantity, only the real part of the velocity is used indicated by the formulation in Eq. (S9). In accordance with [9], a Womersley number of $\alpha=7$ and velocity amplitude of $U_{0}=0,1848\text{ m s}^{\text{-1}}$ (Reynolds number $\mathrm{Re}=U_{0}\cdot2a/v=100$) are used. At the outflow (i.e., the right cross-sectional area) a zero-pressure boundary condition is applied.

As airways can be approximated as almost straight compliant tubes in a first sense, this benchmark setup is deemed suitable to quantify the accuracy of the used computational fluid dynamics methods for high-frequency oscillatory pulmonary flows.

| **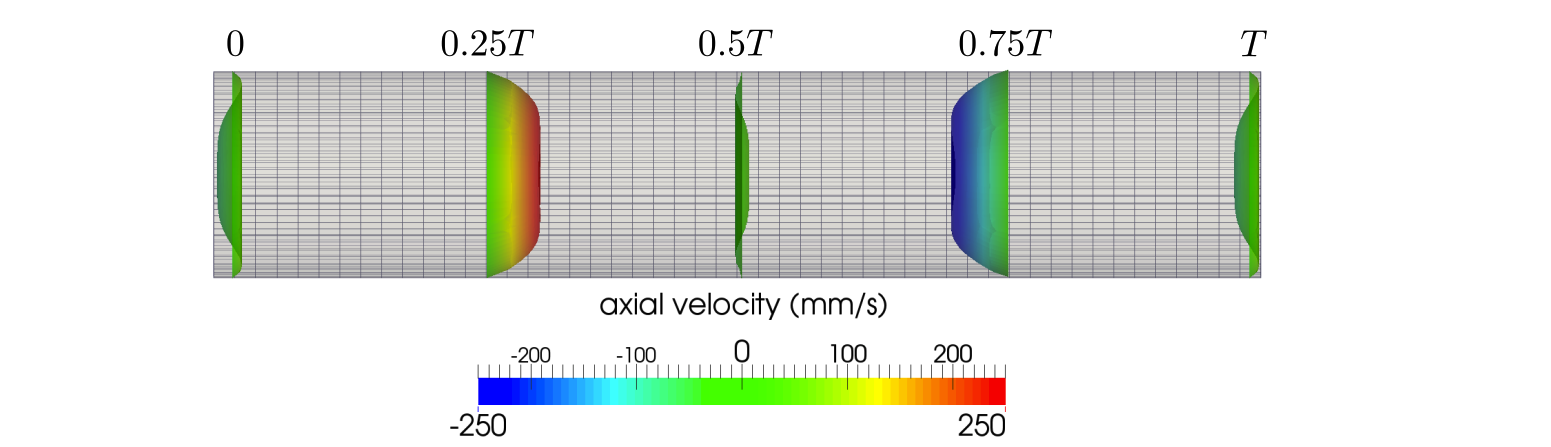** |
| --- |
| (a) |
| **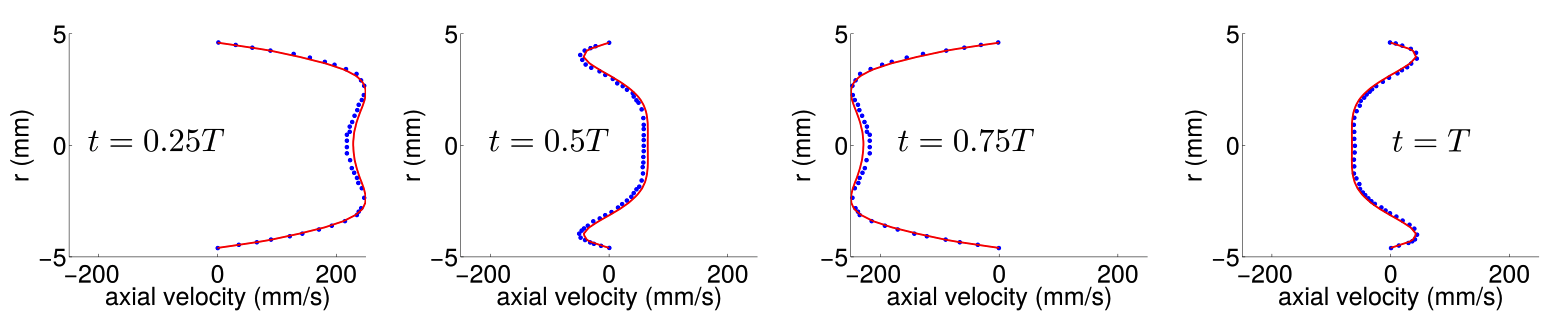** |
| (b) |
| **Fig. 3.** Verification setup consisting of a straight tube with an imposed Womersley profile  ($\alpha=7$, $\mathrm{Re}=100$) at the inlet (left). Fig. 3a shows axial velocity profiles throughout the tube at different points in time where $\times T$ indicates multiples of the time constant $T$. Fig. 3b compares velocity profiles computed in this study (solid red line) with the reference solution by Choi et *al.* [9] (blue dotted line). |

Fig. 3 shows the results of fluid flow for the validation setup described in Section 2.6. In Fig. 3a, the velocity profile is visualised over a complete oscillation cycle. The profile is constant throughout the axial direction of the tube and the different visualised cross sections refer to different points in time of the oscillation cycle. In Fig. 3b, the velocity profiles computed in this study are compared to the results reported by Choi et *al*. [9]. It becomes visible that both solutions agree well and that the Womersley solution inside the straight tube is propagated correctly from the inflow throughout the domain both in the inspiratory phase ($0\leq t\leq0.5\cdot T$; first two profiles) and in the expiratory phase ($0.5\cdot T\leq t\leq T$; last two profiles). At the points of flow reversal ($t\approx0$,$t\approx0.5\cdot T$ and $t\approx T$) backflow phenomena (i.e., negative velocities at positive net flow) are correctly represented. The high accuracy of the solution indicates that the methods presented in this work are suitable for the investigation of HFOV dynamics as they can reproduce the reference solution for this generic scenario.

**S4. Temporal evolution of airflow patterns in the preterm infant**

Temporal evolution of airflow patterns mechanisms detected by the computational lung model and their location in the anatomy of the preterm infant. The movie shows the time span during two breaths. The inspiratory phase ranges from $\left(n\cdot100\right)\text{ ms}\leq t\leq\left(n\cdot100+33\right)\text{ ms}$ and the expiratory phase from $\left(n\cdot100+33\right)\text{ ms}\leq t\leq\left((n+1)\cdot100\right)\text{ ms}$. To illustrate the emerging turbulence during the inspiration phase, λ_2_ iso-surfaces as a widely used criterion for turbulence are visualised in the larger airways in Fig. 2.

**S5. Temporal evolution oxygen concentration in the preterm infant**

Temporal evolution of oxygen transport during a single HFOV cycle. The visualisation refers to the fourth ventilation cycle and annotated time starts at the beginning of the fourth cycle. The inspiratory phase ranges from $\left(n\cdot100\right)\text{ ms}\leq t\leq\left(n\cdot100+33\right)\text{ ms}$ and the expiratory phase from $\left(n\cdot100+33\right)\text{ ms}\leq t\leq\left((n+1)\cdot100\right)\text{ ms}$. The region marked in blue shows the fraction of inspired oxygen larger than 0.25 and is coloured according to the visualised scale.

**References**

1. Ismail M, Comerford A, Wall WA. Coupled and reduced dimensional modeling of respiratory mechanics during spontaneous breathing. Int J Numer Method Biomed Eng. 29, 1285-1305 (2013).

2. Tawhai MH, Pullan AJ, Hunter PJ. Generation of an anatomically based three-dimensional model of the conducting airways. Ann Biomed Eng. 28, 793-802 (2000).

3. Menache MG, Hofmann W, Ashgarian B, Miller FJ. Airway geometry models of children's lungs for use in dosimetry modeling.  *Inhal Toxicol*. **20**, 101-126 (2008).

4. Majumdar A, Alencar AM, Buldyrev SV, Hantos Z, Lutchen KR, Stanley H, Suki B. Relating airway diameter distributions to regular branching asymmetry in the lung. Phys Rev Lett. 95, 168101 (2005).

5. Horsfield K, Dart G, Olson DE, Filley GF, Cumming G. Models of the human bronchial tree. J Appl Physiol. 31, 207-217 (1971).

6. Horsfield K, Gordon WI Kemp W, Phillips S. Growth of the bronchial tree in man. Thorax 42, 383-388 (1987).

7. Neumann RP, Pillow JJ, Thamrin C, Larcombe AN, Hall GL, Schulzke SM. Influence of gestational age on dead space and alveolar ventilation in preterm infants ventilated with volume guarantee. *Neonatology* **107**, 43-49 (2015).

8. Geuzaine C, Remacle JF. Gmsh: a three-dimensional finite element mesh generator with built-in pre- and post-processing facilities. Int J Numer Methods Eng. 79, 1309-1331 (2009).

9. Choi J, Xia G, Tawhai MH, Hoffman EA, Lin CL. Numerical study of high-frequency oscillatory air flow and convective mixing in a CT-based human airway model. *Ann Biomed Eng*. **38**, 3550-3571 (2010).

10. Bauer K, Rudert A, Brücker C. Three-dimensional flow patterns in the upper human airways. *J. Biomech. Eng.* **134**, 071006 (2012).

11. van Ertbruggen C, Hirsch C, Paiva M. Anatomically based three dimensional model of airways to simulate flow and particle transport using computational fluid dynamics. J Appl Physiol. 98, 970-980 (2005).

12. Ma B, Bates JHT, Modeling the Complex Dynamics of Derecruitment in the Lung. Ann Biomed Eng. 38, 3466-3477 (2010).

13. Yoshihara L, Coroneo M, Comerford A, Bauer G, Klöppel T, Wall WA. A combined fluid-structure interaction and multi-field scalar transport model for simulating mass transport in biomechanics. *Int J Numer Meth Engng*. **100**, 277-299 (2000).

14. Gravemeier V, Comerford A, Yoshihara L, Ismail M, Wall WA. A novel formulation for Neumann inflow boundary conditions in biomechanics. *Int J Numer Meth Biomed Engng*. **28**, 560-573 (2012).
